# Advances in Plant Metabolomics and Its Applications in Stress and Single-Cell Biology

**DOI:** 10.3390/ijms23136985

**Published:** 2022-06-23

**Authors:** Ramesh Katam, Chuwei Lin, Kirstie Grant, Chaquayla S. Katam, Sixue Chen

**Affiliations:** 1 Department of Biological Sciences, Florida A & M University, Tallahassee, FL 32307, USA; ramesh.katam@famu.edu (R.K.); kirstie.grant@famu.edu (K.G.); 2 Department of Biology, University of Florida, Gainesville, FL 32611, USA; chuwei.lin@ufl.edu (C.L.); chaquayla.katam@ufl.edu (C.S.K.); 3 University of Florida Genetics Institute (UFGI), University of Florida, Gainesville, FL 32610, USA; 4 Plant Molecular and Cellular Biology Program, University of Florida, Gainesville, FL 32610, USA; 5 Proteomics and Mass Spectrometry, Interdisciplinary Center for Biotechnology Research (ICBR), University of Florida, Gainesville, FL 32610, USA

**Keywords:** metabolomics technologies, abiotic and biotic stresses, multi-omics, single-cell metabolomics, spatial metabolomics

## Abstract

In the past two decades, the post-genomic era envisaged high-throughput technologies, resulting in more species with available genome sequences. In-depth multi-omics approaches have evolved to integrate cellular processes at various levels into a systems biology knowledge base. Metabolomics plays a crucial role in molecular networking to bridge the gaps between genotypes and phenotypes. However, the greater complexity of metabolites with diverse chemical and physical properties has limited the advances in plant metabolomics. For several years, applications of liquid/gas chromatography (LC/GC)-mass spectrometry (MS) and nuclear magnetic resonance (NMR) have been constantly developed. Recently, ion mobility spectrometry (IMS)-MS has shown utility in resolving isomeric and isobaric metabolites. Both MS and NMR combined metabolomics significantly increased the identification and quantification of metabolites in an untargeted and targeted manner. Thus, hyphenated metabolomics tools will narrow the gap between the number of metabolite features and the identified metabolites. Metabolites change in response to environmental conditions, including biotic and abiotic stress factors. The spatial distribution of metabolites across different organs, tissues, cells and cellular compartments is a trending research area in metabolomics. Herein, we review recent technological advancements in metabolomics and their applications in understanding plant stress biology and different levels of spatial organization. In addition, we discuss the opportunities and challenges in multiple stress interactions, multi-omics, and single-cell metabolomics.

## 1. Introduction

The development of technologies for acquiring and analyzing multiple datasets has advanced knowledge of biological systems at the molecular level. These technologies have been applied in omics studies, including genomics, transcriptomics, proteomics, and metabolomics. To date, there has been increasing attention given to identifying, quantifying, and characterizing crucial molecules responsible for biochemical events using large-scale analytical technologies. Among them is metabolomics, which studies all the metabolites, considered final products of biological processes in a cell, tissue, or organism. Although plant metabolomics is an emerging field in omics science, a metabolic study in plants can be traced back to 1817, when Bienaimé Caventou first isolated chlorophyll [1]. Since then, the discovery and isolation of alkaloids, phytohormones, and other metabolites have led to a better understanding of plants’ chemical composition and biological processes [2,3]. Metabolites are bio-indicators of plant phenotypes, including physiological and biochemical properties, which reveal gene expression, signaling, and metabolic pathways. Both primary and specialized metabolites are related to the intrinsic functions of plants, including growth, development, and reproduction. Compared to primary metabolites, specialized metabolites do not directly affect the essential functions of plants but enhance survival under biotic and abiotic stresses. All cells from an individual plant have the same genomic information but different metabolomes. Depending on the cell type, metabolomes vary due to internal and external factors, such as epigenetic information and environmental conditions and stresses. Metabolomics has played crucial roles in numerous disciplines, including gene discovery, fingerprinting for taxonomy, enzymatic reactions, and comparisons between agricultural cultivars, food, and traditional medicine [4]. Metabolites reflect the actions of upstream molecules, including genes, transcripts, and enzymes. Hence, identifying and quantifying metabolites from plant organs, tissues, and single cells under various biotic and abiotic stresses offer multiple avenues for understanding the phytochemistry. These metabolite profiles serve as markers to further assess the impacts of various environmental factors on plants and develop protective strategies in agricultural production.

**Figure 1:**
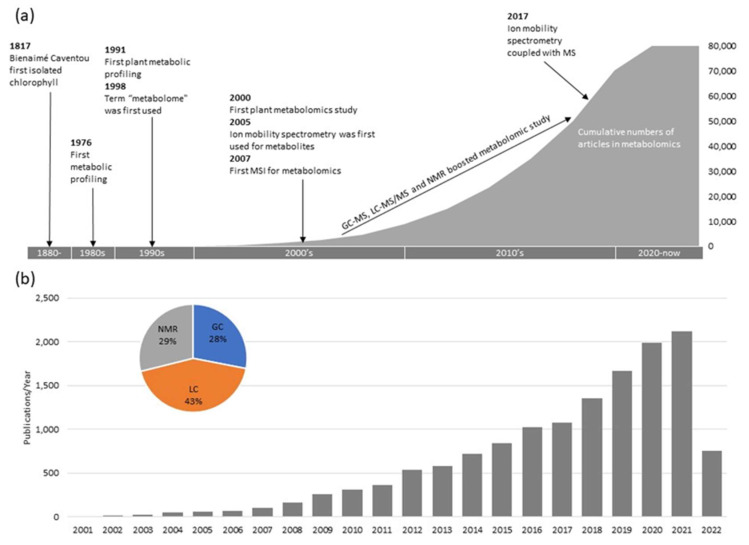
Historic views of metabolomics development and research. Publication numbers were obtained from PubMed search using plant metabolomics as keywords. (**a**) Increased interest in metabolomics research and applications over the past two centuries (MSI, mass spectrometry imaging); (**b**) Publications in plant metabolomics on an annual basis and the technologies employed (insert as a pie chart) in the past two decades. Please note that 2022 is for the first five months only.

Quantitative profiling of metabolites began in the 1980s with the development of gas chromatography (GC), liquid chromatography (LC), and mass spectrometry (MS) technologies [5]. However, it was not until 1991 that the first analysis of plant metabolites was completed using GC. The resulting data revealed the metabolic effects of various herbicides on barley seedlings [6]. Later, high-throughput MS coupled with GC enabled the identification and quantification of metabolites in a broader range [7,8]. This combination allowed for more extensive information, including amino acids, carbohydrates, organic acids, hormones, lipids, and various sugar phosphates. During this time, the LC-based profiling method was reported to detect femtomole level metabolites, such as acyl-CoA esters and flavonoids [9,10]. In the early 2000s, nuclear magnetic resonance (NMR) began to launch its application in plant metabolomics, further increasing the robustness of metabolite fingerprinting and structural elucidation [11,12]. Since then, the MS and NMR technologies have enabled the discovery of more and more metabolites over the past two decades (Figure 1). Today, ion mobility spectrometry (IMS) allows the separation of isobars and isomers, adding a new dimension of separation to modern metabolomic research [13]. 

## 2. Metabolomics Technologies and Advancements

**Figure 2:**
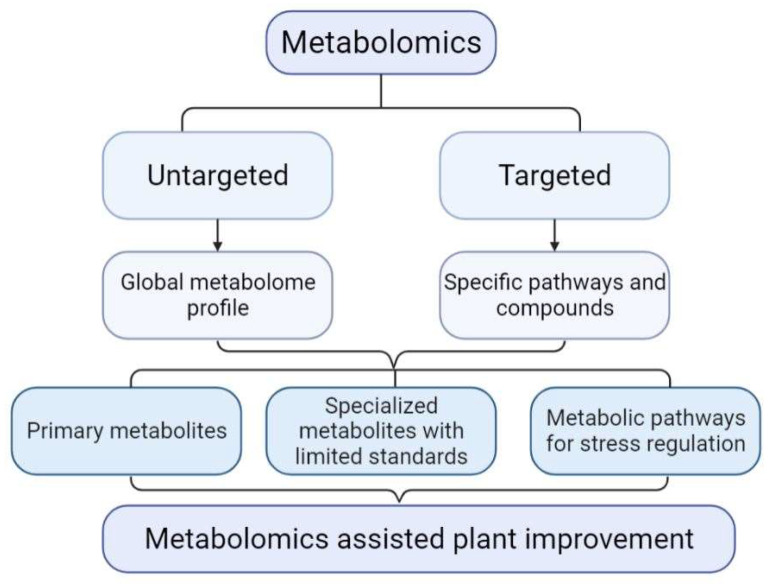
Untargeted and targeted approaches for metabolomic analysis in plants.

Despite the progress in metabolomic studies, obtaining adequate coverage of the metabolome to complement what was achieved for the genome, transcriptome, and proteome remains a significant challenge [14]. This is primarily attributed to the vast diversity in chemical structure and the dynamics of metabolite conversion. Thus far, only about 14,000 metabolites out of potentially a million metabolites in the plant kingdom can be measured, suggesting that advanced analytical methods are needed [15]. Hyphenated technologies involving untargeted and targeted metabolomics (Figure 2) have shown utility in enhancing metabolome coverage and depth [16,17,18]. Untargeted metabolomics, based on high-resolution mass spectrometry, is an extensive compendium of different features in biological samples, e.g., diseased or resistant plants relative to their controls. This method primarily aims to profile all the metabolites, including unknown chemicals. Targeted metabolomics is often based on triple quadrupole mass spectrometry (TQMS), which allows relative or absolute quantification of individual, or a relatively small number of metabolites. Targeted metabolomics primarily focuses on the analysis of specific metabolites with authentic standards. For example, the LC-MS/MS Q Trap was used to profile unique benzoxazinoids, benzoxazolin-2-one, 6-methoxy-benzoxazolin-2-one, and 2-hydroxy-1,4-benzoxazin-3-one in wheat and barley genotypes for weed-suppression [19]. 

The availability of authentic standards often limits targeted metabolomics. Untargeted metabolomics does not have this limitation. However, its disadvantage is a low identification rate of metabolites with known structures. Semi-targeted metabolomics represents a mid-way strategy enabling the quantification of hundreds of metabolites by applying one calibration curve for several metabolites of similar chemical structures [20,21]. An example is field-metabotyping of downy mildew symptoms on grapes, where 32 metabolites were identified at level 1 in the confidence of metabolite identification and 15 more metabolites at level 2 as infection biomarkers [22]. Another method, known as pseudo-targeted metabolomics has emerged. It merges the advantages of both untargeted and targeted metabolomics approaches. This approach automatically defines ion pairs of metabolites for multi-reaction monitoring (MRM) using MM-Ion Pair Finder software for TQMS, without the need of authentic chemical standards [23]. 

Modern analytical instruments for metabolites can be divided into two parts: front-end separation techniques, such as LC and GC, and spectroscopic techniques, such as MS and NMR. These techniques are usually complementary to one another. The combined application of these techniques has been used in numerous metabolomic profiling experiments to answer various biological questions, including metabolites involved in plant stress responses. MS coupled with GC or LC has the advantage of fractionation and separation gained from chromatography and mass analyzer separation gained from MS [24]. While GC and LC separate complex mixtures based on the differential partition of molecules between mobile phase and stationary phase/column, MS and NMR analyze the molecules based on their mass-to-charge ratio in electric fields or resonance frequency in magnetic fields. Here we summarized the development of different instrument technologies for metabolomics applications. 

GC-MS has been popular in detecting volatile metabolites and those amenable to derivatization. Due to the nature of gaseous phase, it enables high ionization efficiency and allows for a reproducible retention time [25]. Because of the usual requirement of chemical derivatization, GC-MS has limited coverage of about 200 metabolites, which encompass mostly the central metabolites [7]. Nevertheless, GC-MS still has unshakable status in its hydrocarbons and volatile metabolite analysis in plant metabolomics [26]. For example, 14 volatile compounds in *Picrorhiza kurroa* roots were extracted with ethyl acetate and butanol, and identified using GC-MS. These metabolites have anticancer and antimicrobial activities [27]. Another study identified 15 volatile metabolites from *Kigelia africana* fruit extracts using GC-time-of-flight (TOF)-MS, but not with LC-MS/MS [28]. Volatile compound yield depends on the extraction solvents used, e.g., aqueous dichloromethane gave the highest yield, followed by methanol and n-hexane [28]. Herein, some recent developments in GC-MS analysis are outlined. First, combining GC-MS with isotopic labeling enabled the study of guard cell glucogenesis [29]. Guard cells were incubated with ^13^C-NaHCO_3_ to allow ^13^C-labeling of glucose, malate, glutamate, and glutamine in guard cells. Quantification of these ^13^C-labeled metabolites provides a detailed view of metabolic flux in glucogenesis. Another development is two-dimensional GC-MS (GC × GC-MS), which provides better separation and peak capacity than conventional GC-MS [30]. Two GC columns with different stationary phases are linked together to achieve better resolution. Two recent examples in plant metabolomics benefited from the use of Pegasus 4D GC × GC-TOFMS system [31,32]. The system applies non-polar capillary column as the first GC column and a polar one as the second GC column, followed by high-resolution TOF-MS. With this system, 400 to 600 metabolites can be detected in *Artemisia afra* tea leaves and Australian indigenous Fruits [31,32]. GC × GC-MS generates thousands of peaks with better resolution and higher capacity to obtain quantification data. However, about 70% of the peaks cannot be interpreted due to lack of experimentally derived databases [33]. Another high-resolution GC-MS system, known as GC-Orbitrap-MS, was introduced in 2014 [34]. An Orbitrap analyzer was used to replace the single-quadrupole commonly found in GC-MS. Although the high-resolution data only slightly improved conventional library matching, the GC-Orbitrap-MS showed clear benefits of sensitivity and accurate annotation of unknown metabolites, and enhanced metabolome coverage [25]. To date, GC-Orbitrap-MS has been used in multiple metabolomic studies involving bacteria, corpses, and human plasma [35,36,37,38]. In a couple of plant metabolomic studies on tomato (*Solanum lycopersicum* L.) and black pepper (*Piper nigrum* L.), GC-Orbitrap enabled identification of biomarkers for processing authentication and quality control of fruits [39,40]. In summary, GC-MS is widely used in certain areas of plant metabolomics, e.g., fruit flavor and ripening. The recently developed GC-Orbitrap-MS will find more utility as the community improves the high-resolution GC-MS databases. 

To date, LC-MS/MS is still the most widely used platform for metabolomics research because of its versatility and high metabolome coverage. Ultra-high pressure liquid chromatography (UPLC) has had widespread applications in plant metabolomics in the last decade due to its high peak capacity and separation efficiency [16,17,18,41]. Although regular flow and microflow LC systems have been widely used, nanoLC (nLC) development has shown great potential in metabolomics [42]. The performance of nLC depends on a miniaturized column with an inner diameter between 10 to 100 μm, when compared with the 1 to 4.6 mm microflow or regular flow columns. The nLC is challenged by its delicate size and lack of robustness. It is sensitive to extra-column dead volume, which has to be minimized to reduce the impact in separation efficiency. In addition, it is liable to column clogging, and low spray stability, which require careful sample preparation and instrument operation skills. Of course, nLC-MS has many advantages. Chromatographic dilution and ion suppression are reduced because of the low flow rate, contributing to high sensitivity of nLC [43]. In addition, less sample is consumed, enabling analysis of samples with limited quantity, such as single-cell samples. Furthermore, low dispersion and flow resistance to mass transfer also contribute to increased column efficiency. During nano-electrospray (nESI), a decrease in evaporative volume leads to a rise in the desolvation rate, allowing more metabolite ions to generate and enter MS [44,45]. Comparative results from nLC-MS and regular LC-MS showed that the former provided a much higher metabolite coverage in human urine, sweat and glioma cells [46,47]. Although nLC-MS/MS has become routine in proteomics research, there are only a few nLC-MS applications in plant metabolomics. In one such example, the nLC-MS technology separated and quantified different cannabinoids in hemp inflorescences [48]. Compared to rosette leaves, hemp inflorescences are of limited quantity. In this case, 0.25 g of inflorescence samples was used for cannabinoid extraction and metabolomic analysis. With the development of nLC-MS, metabolomics can be applied to samples with limited quantity. 

Another advancement is the application of ion mobility spectrometry (IMS) as another dimension of metabolite separation. IMS separates molecules based on their mobility in a drift gas related to the shape, size, and charge of ions. The collision cross-section (CCS) value is inversely associated with ion mobility, the average spatial size, and the shape of a molecule in the gas phase. CCS value is highly reproducible and has become a new standard for molecular analysis. IMS coupled with LC-MS/MS provides the fourth dimension of separation in addition to retention time, precursor *m*/*z*, and product ion *m*/*z*. In addition to drift tube ion mobility spectrometry (DTIMS), field asymmetric waveform ion mobility spectrometry (FAIMS), and travelling wave ion mobility spectrometry (TWIMS), trapped ion mobility spectrometry (TIMS) is a new IMS method. Unlike DTIMSand TWIMS, TIMS holds ions in a parallel gas flow, rather than driving ions through a long drift column. It shortens the analyzer size (~5 cm) and increases the analyzing speed (over 100 Hz). Compared with FAIMS, TIMS provides high resolving power and peak capacity. The first TIMS CCS library of 146 plant metabolites from *Medicago truncatula* was recently reported [13]. Considering the existence of hundreds, and even thousands, of plant metabolites, the plant CCS libraries need to be expanded. Current metabolite databases and MS libraries such as NIST and METLIN have started to include CCS values of metabolites. In addition, TIMS has the potential to separate isomeric and isobaric molecules, which has attracted great interest in advanced metabolomics applications, e.g., in-depth lipidomics [49]. In addition to TIMS, cyclic IMS (cIMS) and structures for lossless ion manipulation (SLIM) IMS are two new technologies that have been commercialized [50,51]. The cIMS contains a pre-array store, which allows trapping fragmented ions before entering TOF, enable IMS^n^. In SLIM, ions can be quickly separated and quantified in a lossless manner, increasing the sensitivity and resolution of ion mobility separation. With more experimental standards being included in different metabolite databases, IMS will become increasingly popular in the future.

NMR has been the gold standard for molecular structure elucidation and is a complementary analytical technique to MS. Compared to MS, NMR is nondestructive and provides a more detailed view at the atomic level. It can be used for real-time metabolomics in living samples. NMR is known to be less sensitive than MS. It requires metabolites at microgram/micromole levels, while MS can detect metabolites at femtomole or even attomole levels. The most common method used for analysis in plant metabolomics is 1D (1H) NMR, e.g., metabolomics of abiotic and biotic stresses [52,53,54], novel compound identification [55], and evolutionary research [56]. This method, 1D NMR, allows high-throughput analysis of hundreds, or even thousands, of samples, since it takes only a few minutes per sample. However, reliable metabolite identification in complex mixtures is achieved with 2D NMR methods (e.g., ^13^C-^1^H), which resolve peak overlap in crowded regions of the spectra [57]. Recently, LC-NMR has shown utility as an efficient tool in analyzing natural products from plant extracts, such as isoflavonoids from *Ancistrocladus guineensis* leaf, phytosterols from *Urtica dioica* root, and limonoids from *Swietenia macrophylla* seed [58]. Today, with the increasing availability of GC/LC-MS, a combination of GC/LC-MS and NMR may provide extensive coverage of metabolites in plants [59]. A recent study of *Cassiae semen* seeds utilized both LC-MS and NMR, and identified 231 metabolites using LC-MS and 41 using NMR [60]. Please note that front-end GC or LC separation is not always necessary. For example, direct analysis in real-time mass spectrometry (DART-MS) uses ambient ionization to directly analyze solids, liquids, or gases by exposing them to the open-air space between the mass spectrometer inlet and the ionization source [61]. DART-MS-derived fingerprints have been used to distinguish crops cultivated through organic farming from conventional farming [62]. 

High-throughput instruments with lower sample requirements and higher sensitivity contribute to the development of single-cell metabolomics. Over the past five years, single-cell sorting techniques such as flow cytometry, free-flow electrophoresis, and laser microdissection have been developed based on cell size, mass, and labeling [63]. Most of the methods have been applied to animal cells. In plant cells, protoplasts can be isolated for single-cell metabolomics studies [64]. However, protoplast preparation involves enzymatic reactions to remove the cell walls. It is not only time-consuming, but also may alter the chemical properties of plant cells. Direct sampling from plant cells is a trend in single-cell metabolomics. Probes with micrometer tips attached to ESI (PESI) enables direct sampling from living plant cells. Tips penetrate the cell, directly sucking out the contents of the cell. Extracts are sent to ESI-MS without any pre-treatments. Usually, the location of the tip will be monitored under a microscope. With this technique, single-cell metabolomics has been applied to epidermal cells [65], mesophyll cells [66], trichome cells [67], and phloem associated cells [68]. Recently, PESI has been further developed into pressure-PESI (PPESI), utilizing a cell pressure probe [67]. The probe is used to assess cellular properties, including water potential, cell wall elasticity, and plasma membrane hydraulic conductivity. PPESI can distinguish live cells before sampling based on their water status. The cell pressure probe is usually a quartz capillary that can take cellular content by picolitres. The use of internal electrodes in capillary holders was recently introduced and has vastly improved the detection sensitivity by providing a high voltage on targeted cell sap. This technique is also referred to as picoPPESI [69]. 

Mass spectrometry imaging (MSI) is a powerful tool that provides in situ analyses of spatial localization and relative abundance of metabolites. Unlike other biomolecular imaging methods, MSI does not require labeling and can simultaneously analyze a wide range of metabolites [70]. Matrix-assisted laser desorption ionization (MALDI) is the most popular MSI method since it covers many molecules. It has been applied to tomato fruits [71], green algae [72], and maize roots [73]. To image with MALDI, sample preparation requires delicate freezing, sectioning, and mounting [74]. For fruit, leaf, and root samples, fresh tissues need to be embedded into a cryo-mold and sectioned using a cryostat [71,73]. The tissue sections (e.g., 35-µm-thick tomato fruit sections and 20-µm-thick maize root sections) are put on glass slides and sprayed with different matrixes. Since green algae are free-living cells, they can be directly spotted onto gold microchips, and dried for MALDI analysis [72]. MALDI MSI made it easy to view metabolite distribution and abundance in tissues and cells. However, it is challenging to use MALDI for single-cell imaging of plants because of matrix crystals and the limitation of laser size. Laser-ablation electrospray ionization (LAESI) is a newly developed MSI approach. Unlike MALDI, LAESI can analyze a sample in atmospheric conditions, and requires little to no sample preparation (e.g., matrix-free) [75]. With the fine glass fiber tip, LAESI can produce a laser spot size of 10 to 50 µm, achieving single-cell analysis in root nodule cells and leaf epidermal cells [76,77]. To date, single-cell metabolomics by LAESI is limited by its low efficiency of targeting cells. Development of automated image processing may enable high throughput single-cell metabolomics with LAESI. With the advancements in metabolomics technologies, scientists have gained an improved understanding of different plant biological processes, e.g., plant stress responses.

## 3. Metabolomics Applications in Plant Stress Responses, Multi-Omics and Single-Cell Biology

The metabolome is highly dynamic, and metabolomics has shown utility in capturing constant metabolite changes that reflect cellular signaling and metabolic processes, organismal phenotypes, and traits. For example, bioactivity profiles of *Fucus vesiculosus* sampled monthly for one year indicated that many metabolites were found to vary with the sampling time; phlorotannin total ion count (TIC) was highest in summer, while chlorophylls, lipids, and carotenoids peaked in winter and spring [77]. Untargeted metabolomics revealed metabolic plasticity of xylem lignification in hybrid poplar trees with different genetic backgrounds, suggesting genotype-specific metabolism linked to specific phenotypic traits [78]. Also, the primary and specialized metabolite profiles of citrus rootstocks explained their scions’ tolerance to pathogens [79]. GC-MS metabolomics demonstrated that cuticular wax composition and crystal microstructure variation exist among tissues and cultivars of common wheat [80]. Another metabolomic study in pepper revealed that the shift of polyphenols occurred during fruit ripening in different fruit tissues and their abundances were cultivar-dependent [81]. Herein, we focus on the metabolomics of plant stress responses, multi-omics, and single-cell biology.

### 3.1. Diversity of Plant Metabolites as a Result of Ecological Adaptation

Plants produce a vast diversity of approximately one million metabolites, the majority of which are still unknown [82]. Primary (central) and specialized metabolites are synthesized continuously via complex biochemical reactions. Primary metabolites are essential for the biosynthesis of lipids, sugars, and amino acids. They mediate the tricarboxylic acid cycle, glycolysis, and photosynthesis, affecting plant growth and development. Variations in the synthesis of primary metabolites may lead to perturbation of photosynthesis, respiration, and imbalanced osmotic adjustment in plants. While the reactions of primary metabolism are conserved, these pathways serve as precursors to produce specialized metabolites, such as flavonoids, phenolics, carotenoids, alkaloids, glucosinolates, and phytic acids in different plant lineages, deemed previously non-essential in plant growth and development. Thus, they used to be called secondary metabolites, also known as natural products. Specialized metabolites are often the products of the plant responses to different abiotic and biotic stress conditions, such as high temperature, chilling, drought, salinity, and insect/pest attack. These specialized metabolites are adapted to the multitude of environmental challenges over time. The adaptations can be attributed to the evolution of specialized metabolite pathways. Their products are highly diverse in their chemical structure, in parallel with genetic diversity in the biosynthesis of these specialized metabolites [83]. It is thought that gene duplication led to a high degree of diversity among the specialized metabolites [84]. 

### 3.2. Dynamics of Plant Metabolites in Response to Stresses

**Figure 3:**
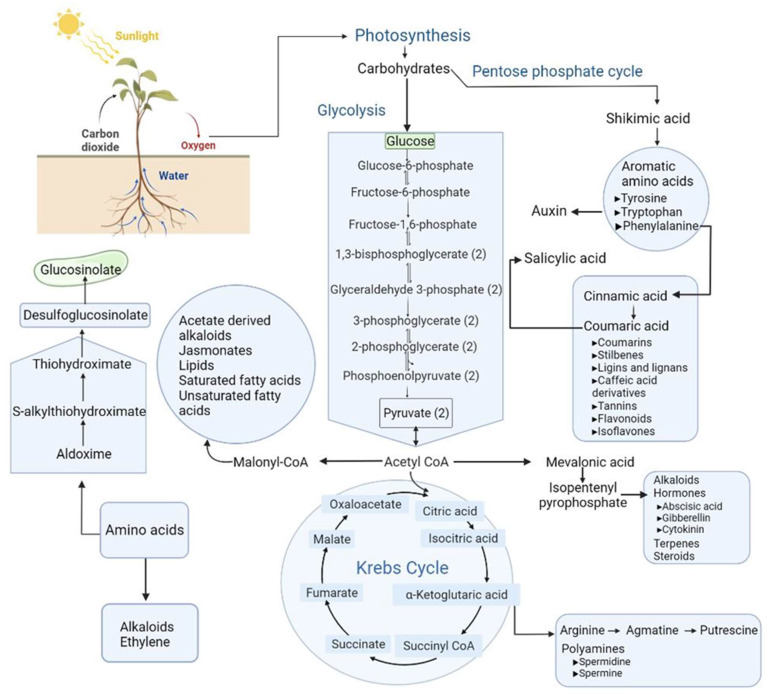
Metabolic pathways and connections depicting primary metabolites and their derived specialized metabolites in response to abiotic and biotic stresses.

Abiotic and biotic stresses have been a constant threat to crop production affecting plant growth and development, including germination, nutrient uptake, photosynthesis, yield, quality, and primary and specialized metabolites (Figure 3). Plants produce more metabolites than animals or microbes [85,86]. As sessile organisms, plants have evolved mechanisms to respond and adapt to changing environmental conditions. One of the mechanisms is the production of various defense-related metabolites representing the specialized metabolites, including flavonoids, alkaloids, and glucosinolates. The plant responses also alter the signaling pathways, lipids, phytohormones, protein kinases, reactive oxygen species (ROS), and redox homeostasis [87]. Therefore, understanding the molecular and chemical processes involved in signaling and metabolic processes (e.g., synthesis of defense metabolites, osmoprotectants, and cell wall strengthening components) is crucial for understanding the plant responses to the specific stress. It is also necessary to scout the relationships between the levels of metabolites, enzyme activity, and transcript levels of known and unknown functions. Variations in the concentration of numerous metabolites may give mechanistic indications for biochemical networks that define plants’ phenotypic and physiological feedback to environmental fluctuations [88].

#### 3.2.1. Abiotic Stresses

Abiotic stresses continually challenge plants, and global climate change has further increased the adverse effects of these abiotic stresses, including the spatial variation in ozone on crop yield and food security [89]. Studies suggest perturbation of the genetic regulatory system due to the progression of climate change [90]. The prime objective of investigating metabolic variations under abiotic stresses is to profile different metabolites that permit signaling and metabolic adjustment, restore plant homeostasis, and adapt to the conditions. Herein, we discuss metabolomic profiles and their dynamic changes as indicators and responses to the adverse effects of various abiotic stresses. Primarily, we present the metabolomics studies in model plants (see Table 1 and refer to Supplemental Table S1 for comprehensive coverage of plant metabolomics studies to stress). 

##### Drought, Flooding and Heat Stress

Plants adopt several physiological modifications in drought, including leaf surface reduction and increased nutrient uptake through roots. In this process, plants synthesize many metabolites in response to drought as a defense mechanism. Targeted LC-MS-based profiling provided high sensitivity and specificity analysis of specific metabolites in leaves under water-deficit. The data indicated a significant breakdown of specific galactolipids and accumulation of unsaturated fatty-acid derivatives and diverse jasmonates (JAs) in *A. thalaiana* [93]. Water stress influenced the biosynthesis of different polyphenol classes in grape skins, which reflects the cultivar differences in metabolic responses to water deficit [113]. Metabolomic studies in peanuts showed that biological nitrogen fixation in nodules from the drought-tolerant cultivar efficiently accumulated protective metabolites, including trehalose, proline, and gamma-aminobutyric acid (GABA). After re-irrigation, the drought-stressed nodules of a tolerant cultivar reached the metabolic state of unstressed plants. In contrast, a drought-sensitive cultivar failed to re-establish metabolism upon re-hydration and displayed growth inhibition [114]. 

Waterlogging hinders crop growth due to limited supply of CO_2_ and oxygen, which hampers photosynthesis and respiration [115]. NMR-based metabolomic profiling of soybean indicated increased synthesis of primary and specialized metabolites in response to waterlogging. For example, the concentrations of phosphoenolpyruvate, nicotinamide adenine dinucleotide (NADH), glycine, and gamma-aminobutyric acid were increased under waterlogging conditions, leading to modulation of the urea cycle in the soybean root tips [116]. Upon flooding, wheat cultivars showed initial carbohydrate consumption, associated with the expression of genes encoding sucrose and fructan catabolism enzymes, linked to submergence tolerance [117]. The tolerant wheat cultivar survived extended periods of low shoot carbohydrate levels by accumulating most amino acids. 

Drought often co-occurs with heat stress. Under heat stress, *M. truncatula* synthesizes many primary and specialized metabolites, such as rhamnose, putrescine, myoinositol, 2-ketoisocaproic acid, arachidic acid, allantoin, and alanine. Increased pipecolate and tryptophan as well as decreased sugar and sugar alcohols were observed [118]. However, in wheat, higher levels of sucrose and glucose-1-phosphate were detected under heat stress compared to the control [119]. The study of drought tolerant and sensitive soybean (*Glycine max*) under drought stress found that aspartate, 2-oxoglutaric acid, myo-inositol, pinitol, sucrose and allantoin in the leaves, and 2-oxoglutaric acid and pinitol in the nodules were differentially changed in different genotypes [120]. However, heat-tolerant soybean seedlings exhibited high levels of 1,3-dihydroxyacetone, ribose, glycolate, flavonoids, ascorbates, and tocopherols. Ascorbated and tecopherols act by attenuating the deleterious effects of heat-induced ROS damage during seed maturity at high temperatures [121]. In summary, although drought and flooding seem to be contrasting stress conditions, the metabolic changes are not merely opposing, but rather sophisticated (e.g., both involving increased amino acids). Heat-induced metabolic changes are different from those induced by drought. Species variation in the different stress responses are also noted.

##### Salinity Stress

Soil salinity alters ion uptake, causing metabolic and osmotic imbalance, which results in poor plant growth and yield. Imbalanced Na^+^ ion concentrations cause ion toxicity to hamper nutrient and water uptake in high salinity conditions. Under salinity, sorghum (*Sorghum bicolor*) plants exhibited altered growth parameters, water relations, ions, compatible solutes, polyamines, and metabolite profiles identifying the tolerant sorghum phenotypes [122]. Metabolite profiling of a desert grass *Aeluropus* showed increases in free amino acids and organic acids, while the polyols were decreased in both shoot and root tissues under salinity [123]. Salinity also caused a significant shift in the lipid distribution in young developing barley root tissues in a zone-specific manner and distinct changes in phytohormones in the roots [107,108]. In three rice cultivars with different salinity tolerance, salt stress altered the levels of a range of sugars and organic acids, but it did not affect the shoot length, root length, and root fresh weight of salt-tolerant cultivars [106]. Accumulation of specialized metabolites and essential oils was positively modulated by salinity stress in *Ballota nigra* [124]. Untargeted metabolomics of three closely related species of plant halophyte, *Sesuvium* revealed that each species exhibited distinct metabolic adaptation to acquire the salt tolerance. Specialized metabolites such as delphinidin, cyanidin, and hesperidin were found to play a role in osmotic regulation and osmotic stress-induced oxidative imbalance in the closely related species, *Salicornia* *brachiata*, *Suaeda maritima*, and *Sesuvium portulacastrum* [125]. In *S. brachiata* under salt stress, tetrahydrobiopterin and folate cofactors were induced, indicating the role of one-carbon metabolism in response to salt stress. In *S. portulacastrum*, carotenoids and pterins increased, while tricaffeoyl spermidine decreased in *S. maritima* under salinity. Clearly, osmotic regulation is important for salt stress tolerance. Metabolomics studies have revealed changes in many other metabolites, and their exact functions remain to be determined.

##### Metal, Atmospheric, and Nutrient Stresses

Metal stress prompts many changes in plants, including biochemical and physiological alterations, stabilization of protein structure, and perturbation in metabolism, growth, and development. Metal ion stress led to hyperaccumulation of metabolites in *Brassica rapa* leaves and roots as a metal tolerance mechanism. The metabolites include glucosinolates, hydroxycinnamic acids, and primary metabolites such as carbohydrates and amino acids, according to the types of the metal and concentrations [126]. For example, there were more metabolomic changes in Cu and Fe treatments than in the Mn treatment. Similarly, metabolomics of hydroponically grown sunflower roots and leaves revealed the elicited production of biologically active isocoumarins, terpenoids, sesquiterpenes, and their lactones phomallenic acid A, benzofurans, phenylpropanoids, and iridoid glycosides in response to chromium metal stress [127]. 

Metabolomics has been applied to reveal molecular processes in strawberries after harvesting under three atmospheric conditions: ambient, CO_2_-enriched, and O_3_-enriched atmospheres [128]. Metabolic shifts and changes helped to elucidate the involvement of metabolites in fruit organoleptic properties, nutritional properties, and stress tolerance during the postharvest treatments. Additionally, ethanol, ethyl, butyl, 1-methyl ethyl acetates, ethyl octanoate, hexyl and methyl butanoates, GABA, succinic acid, and bis-(HHDP)glucose were identified as prospective biomarkers found to assist in predicting strawberry organoleptic corrosion. In this study, the use of an ozone-modified atmosphere positively impacted fruit aroma by reducing fermentative compounds and increasing methyl butanoate and fruit tolerance to the abiotic stresses generated by postharvest cold storage redirecting metabolism towards the accumulation of protective metabolites. The results open up new opportunities for identifying new genotypes with increased fruit shelf-life and fruit quality stability.

Nitrogen (N) is the essential element of nucleic acid, amino acids, proteins, and specialized metabolites. Metabolic fingerprinting in tomatoes under N and phosphorus (P) deficiencies observed that N stress decreased the concentrations of organic acids and amino acids while triggering the synthesis of soluble sugars [129]. Recently, Ghosson et al. [130] studied the effects of S stress on the metabolites in the roots and leaves of barley. The untargeted metabolomics revealed the changes in different amino acids, organic acids, and S-responsive metabolites under S stress, suggesting discriminant biomarkers following their quantitative validation. Large-scale metabolomics to compare the component variant of wheat under different N levels confirmed the effect of low N stress on the metabolism and revealed the accumulation of flavonoid biomarkers, including isoorientin iso-vitexin, methylisoorientin-2″-O-rhamnoside, and iso-schaftoside [131]. Sorghum roots grown under low N showed an accumulation of trehalose, quinic, and shikimic acids, essential in N storage and synthesis of aromatic amino acids [132]. Drought, water, heat, and changes in atmospheric and nutrient conditions are among the major outcomes of global climate change. The metabolic changes during these stresses drive us to suggest that breeding crop cultivars tolerant to these stresses must be based on their responses to multi-stresses. Therefore, future studies need to focus on metabolomics of plant multi-stress responses and tolerance (see Section 3.3 and Section 3.5).

#### 3.2.2. Biotic Stress Resistance in Plants

The emergence of new diseases and the high genetic variability of phytopathogens and herbivores call for solutions to combating these agents. Recent plant microbiome studies have revealed the functions of beneficial microbes in the phyllosphere and rhizosphere. Metabolomic understanding of the signaling and metabolic responses in plant interactions with biotic factors will help identifying metabolites (e.g., hormones and their crosstalk) important in plant defense against pathogens and herbivores, as well as its association with beneficial microbes. 

##### Metabolomics of Plant-Microbe Interactions

Plants are constantly in contact with microbes in the rhizosphere and phyllosphere throughout their life cycle [133]. Information on the metabolic changes induced during tripartite interactions (plant, toxigenic microbes and beneficial microbes), hereafter defined as plant-microbe interactions, would significantly improve our understanding of molecular signaling and metabolic exchanges between plants the microbes. The plant-microbe association can trigger defensive pathways against pathogen attacks and produce resistance by inducing highly diversified metabolites. These pathways include the biosynthesis of complex plant metabolites and their systemic signaling in the cell. For example, a plant growth-promoting fungus (PGPF) *Trichoderma harzianum* has a synergistic effect on phosphorous (P) uptake by *Zea mays* from compost. The increases of phosphatidylcholine and choline are related to P absorption in the plant, and enhancement in total phenols, SOD, PRX, and PAL enzymes to regulate the host defense in leaves supported its photosynthesis [134]. However, the combination of *Trichoderma* with inorganic fertilizer showed less effectiveness in promoting growth and reduced plant biomass and N content. The metabolic responses varied with the type of P fertilizer and the amendments in combination with the *Trichoderma* inoculum. Thus, metabolome data provide important evidence of the positive effects of the combined *Trichoderma* activity in sustaining plant growth and a possible alternative to P application.

The impact of the beneficial bacteria *Paraburkholderia phytofirmans* PsJN strain on grapevine specialized metabolism showed that PsJN was able to induce locally (in roots) and systemically (in leaves) phenolic acids and their derivatives [135]. Methylobacterium regulates the production of phytometabolites connected with flavor by metabolizing plant host metabolites and some volatile organic compounds [136]. Studies have shown that root exudates containing organic acids (e.g., nicotinic, shikimic, salicylic, cinnamic, and indole-3-acetic acids) could influence the root-microbe interactions [137,138]. The amalgamations of plant exudation and microbial nutrient traits interact to produce unique microbial community assemblies [139]. Further exploration of metabolic and molecular changes connected with *Bacillus velezensis* to mediate abiotic stress tolerance in wheat indicated that *Bacillus* improved wheat survival exposure to heat, cold/freezing, or drought stress [140]. Durum wheat inoculated with another beneficial microbe *Pantoea agglomerans* showed that considerable growth of wheat seedlings, increased chlorophyll content, lower accumulation of proline, and favored K^+^ accumulation in the inoculated plants compared to Na^+^ accumulation in non-inoculated plants [141]. *A. thaliana* infected with pathogen *Pseudomonas syringe* was examined to understand the changes in hormone and redox metabolites, such as in systemic leaves, using a targeted metabolomics strategy to identify and investigate potential functions of specific hormones and redox-related metabolites in systemic acquired resistance (SAR) during plant immune responses [121]. Pang et al. [142] developed a stable isotope labeling method to make bacterial metabolites heavier, so that the same metabolites may be easily differentiated from those in plant cells using MS. It allows the investigation of metabolomic responses from both partners in the plant-microbe interactions. A recent untargeted metabolomics study revealed that root colonization of growth-promoting fungi *Trichoderma harzianum* and *Rhizophagus irregularis* elevated the steroidal glycoalkaloid levels and modified the fatty acid amides and carnitine-derived metabolites in tomato shoots. As a result, the metabolomes of feeding insects were altered, impairing their development and metamorphosis [143]. This study is a great example highlighting the utility of metabolomics in understanding plant interactions with microbes and herbivores. 

##### Metabolomics of Plant-Herbivore Interactions

Phytochemical diversity reflects the plant chemical landscape, chemical ecology, and co-evolution of metabolites, indicating the adaptive response of plants to herbivory [144]. Herbivores adapted to the specific habitat of plant communities may produce unique effects on host plants, including specialized metabolites. For example, an increase in phenolic acids and flavonoids (especially quercetin) has been observed in white cabbage (*Brassica oleraceae* L. *var. capitata*) upon infestation by cabbage butterflies (*Pieris brassicae* L.) and flea beetles (*Phyllotreta nemorum* L.) [145]. The results showed that these species had different effects on the levels of superoxide (a ROS) in predated leaves. The exposure to oviposition by butterflies and subsequent feeding by newly hatched caterpillars did not manifest in higher total phenolic content than predation by flea beetles. The leaves after flea beetle attack also showed an increase in ascorbic acid, an important redox metabolite and ROS scavenger in plant cells. Kariyat et al. [146] showed that 3-deoxyanthocynadin (a flavonoid) present in wild-type sorghum caused significantly higher mortality and reduced population growth in corn leaf aphid (*Rhopalosiphum maidis*) when compared to null mutants devoid of this flavonoid. Purple corn’s polyphenol-rich pericarp extract negatively affected tobacco hornworm growth, development, and adult fitness traits (*Manduca sexta* L.) [147]. Consistent with these observations, it has been well-documented that different groups of polyphenols collectively protect most plant species against a wide range of insects. Plants have evolved natural defense systems to overcome insect pests and pathogens by producing natural repellents known as glucosinolates. There are three types of glucosinolates, aliphatic, indolic, and benzoic glucosinolates produced in Brassicales. Upon insect attack, the glucosinolates and their hydrolyzing enzymes (myrosinases) are triggered to release toxic degradation products to deter generalist insects, while specialists are attracted by some of the degradation products [148]. Sugars and benzoxazinoids were also found to guide the foraging cues for certain herbivores [149]. The distinctive and combined roles of primary and specialized metabolites reveal herbivore foraging strategies linking to their performance and survival. The alarming rise in pesticide resistance in insects warrants the need to identify natural sources of resistance in crop plants. Untargeted metabolomics has identified natural variations in *Capsicum* resistance to thrips [150]. Monomeric capsianosides and dimer acyclic diterpene glycosides were associated to thrips resistance, while sucrose and malonylated flavone glycosides were related to susceptibility in *C. annum* and *C. chinesis* accessions. These results may inform the development of natural pesticides for herbivore defense. 

##### Metabolomics of Hormonal Crosstalk

Plant hormones play a crucial role in regulating the interactions between plants and their abiotic and biotic environments. Over the past decades several metabolites, e.g., JA, salicylate (SA), strigolactone, brassinosteroid, and melatonin have been recognized as new families of plant hormones besides the classical hormones of abscisic acid (ABA), GA, auxin, cytokinin and ethylene (ET). These hormones trigger specific molecular pathways, which are integrated in a network of interactions often called hormone crosstalk [151]. Hormone crosstalk affects genetic information flow, protein stability, metabolite changes, and hormone homeostasis, thus playing an essential role in plant responses to diverse pathogens, herbivores, and environmental conditions [152]. For examples, integrative metabolomics and proteomics revealed the intricate balance between JA and SA in the MAP kinase (MPK) 4-mediated plant immune response [153]. Increases of both auxin and ethylene caused changes in phenylpropanoid, glucosinolate, and fatty acid metabolism in *Arabidopsis* root metabolome, and the changes correlated negatively with the corresponding transcriptome data [154], indicating post-transcriptional events such as changes in enzyme activity and/or transport processes played a role in the metabolomic changes. Stomatal guard cells have been used as a model system for studying hormone function and crosstalk. Using targeted and untargeted metabolomics, Jin et al. (2013) showed that the effects of different hormones converge on shared downstream components and crosstalk occurs at the downstream level. Stomatal closure triggered by ABA is in fact a result of multiple hormones, including IAA, JA and cytokinin. ABA operates upstream of other hormones to regulate their concentrations [155]. IAA was found to crosstalk with cytokinin and ABA and affect plant–microbe interactions in the maize–*Bipolaris* system. The hormone crosstalk led to reduced plant defense and altered the endophytic fungus from symbiotic to pathogenic [156]. 

The individual and combined signaling effects of ET and ABA identified multiple components of MAPK signaling pathways in ET and ABA signaling, indicating the pivotal role of MAPK pathway in the crosstalk of ABA and ET [157]. In response to ET treatment, accumulation of flavonoids and isoflavones (genistein, daidzein and genistein) was observed. The increase in these metabolites may constitute a response to the generation of ROS, which are common components in ABA and ET signaling. Flavonoids and isoflavones are effective ROS scavengers during oxidative stress [158]. In addition, changes in several classes of lipids including glycerolipids, prenol lipids and phospholipids were detected. They include phosphatidyli-nositol phosphate (PIP), diacylglycerol (DG), phosphatidylglycerol (PG), and phosphatidylethanolamine (PE). Most of these lipids were decreased. In contrast, these lipids were increased in response to ABA treatment. As the lipids play crucial roles in cellular structure and signaling, their opposite changes reflect the antagonistic interaction between the two important hormones. 

Melatonin (N-acetyl-5-methoxytryptamine) is well-known for its sleep-promotion and was first discovered in bovine pineal gland [159]. Since its discovery in plants in 1995, it has been found to be the master regulator of plant growth, development, and stress responses [160,161]. Melatonin has multiple regulatory functions in plants. For example, it modulates the expression of genes related other plant hormones and the metabolism of auxin, ABA, GA, cytokinin and ET. Both melatonin and auxin are derived from tryptophan. Due to the structural similarity, melatonin was shown to act as a plant growth stimulator that promotes seed germination, root growth and shoot growth. It also has antioxidant activity, protecting cellular structures from oxidative damage and delaying senescence. Interestingly, melatonin regulates the expression of ET-related genes, thereby affects fruit ripening and post-harvest processes [162,163]. Last but not least, melatonin functions as a stressor protector in plants. For example, the application of exogenous melatonin resulted in increased salt tolerance in rice plants [164]. Metabolomics revealed increased levels of amino acids, organic acids, nucleotides, endogenous melatonin and its intermediates, gallic acid, diosmetin, and cyanidin 3-*O*-galactoside. Some of these metabolites have antioxidant functions, suggesting the alleviating effects of melatonin via antioxidant pathways to salt stress. In plum seedlings under salt stress, high SA and JA levels were observed [165]. Furthermore, melatonin plays an important role in plant immune responses, together with other plant hormones such as JA and SA [160,163]. How melatonin crosstalk with these and other hormones deserves further investigation. 

### 3.3. Plant Interactions with Multiple Abiotic and Biotic Stresses

In natural environments, plants perceive multiple environmental factors at any given time. It is important to decipher how multiple abiotic and biotic stresses crosstalk in plant cells, leading to changes in phenotype/trait. Drought stress increased most metabolites such as asparagine, isoleucine, leucine, methionine, phenylalanine, proline, tyrosine, valine, sarcosine, and trigonelline in *Vitis vinifera* xylem sap. The increase in these metabolites escalated infection progression of bacteria *Phaeomoniella chlamydospora* or *P. minimum* in plants exposed to water stress [166]. To examine the physiological role of sucrose cycling, Weiszmann et al. developed a kinetic model to simulate the dynamics of subcellular sugar concentrations in *Arabidopsis* under combined cold and high light stress [97]. The resulting model revealed that subcellular reprogramming of invertase-driven sucrose cleavage varies substantially between the natural accessions of *Arabidopsis*, which differ in their cold tolerance levels. In another study, metabolic responses of moss *Physcomitrella* *patens* to abscisic acid (ABA), cold, and salt stress indicated significant changes in the accumulation of several sugars. The metabolic responses provoked by ABA, cold, and salt showed considerable similarities in sugar metabolism [103]. Metabolomics of *Mikania laevigata* and *M. glomerata* showed that the volatiles were qualitatively similar, except for coumarin, which is present only in *M. laevigata*, Different seasons, time of the day, and growth conditions such as temperature, light intensity, and water, significantly affected the composition and intensity of headspace volatiles in both species [167]. The effects of combined chilling and UV-A treatment on the accumulation of phenolic metabolites in *Brassica oleracea* var. acephala demonstrated that UV-A irradiation significantly increased plant growth parameters, including shoot and root dry weight, leaf area, and photosynthetic rate [168]. Although chilling-stress inhibited plant growth, plants under combined cold and UV-A stress maintained similar growth parameters. Treatment with chilling plus UV-A increased ROS levels in kale, stimulated the biosynthesis of specialized metabolites, and induced the accumulation of phenolic antioxidant metabolites. The study suggests that treatment with the optimal level of combined chilling and UV-A could improve phenolic metabolites in kale and its nutritional quality. This potential technology can be adapted to other vegetable crops and medicinal plants. 

In *Haematococcus luvialis*, the combined effect of fulvic acid (FA) and abiotic stresses of high light and low N enhanced the astaxanthin and lipid accumulation. These combined stressors imposed oxidative stress and increased the contents of glucose-1-phosphate, malate, and other metabolites, such as glutathione (GSH), astaxanthin, and lipids [169]. Some cytoprotective metabolites and signal molecules including intermediates in the TCA cycle and Calvin cycle (e.g., succinate, malate, and sugars), melatonin (MT), and some amino acids were increased under FA and abiotic stress conditions. In Australian bread wheat under drought and low N, the highly increased allantoin levels caused induced expression of allantoin catabolic genes, which help regulate allantoin levels. Interestingly, the allantoin levels decreased significantly, and ammonium was liberated through allantoin catabolism when the plants experienced N deficiency [170]. The result indicates that the accumulation of allantoin under drought overcomes its degradation to ammonium, thereby preventing N loss. Thus, allantoin may serve as an internal organic N source to regulate plant N homeostasis under drought and low N stresses. 

As a nitrogen-fixing legume, *Medicago tranuclata* is a valuable forage crop sensitive to abiotic and biotic stresses. The effects of the combined stresses of drought and infection from the soil-borne pathogen, *Fusarium oxysporum*, have not been widely studied. One study investigated metabolite changes due to these combined stresses and identified stress marker metabolites [111]. They include sucrose and organic acids such as citric acid, malic acid, and dehydroascorbate. Under pathogen infection, an increase in flavonoids, sucrose relocation from leaves to roots, an increase in tetrahydroxychalcone (butein), and a decrease in organic acids were observed. Persistent stress-induced changes in metabolites and metabolic fluxes represent metabolic imprints in response to previously occurred stresses, and these imprints can prime the plant’s tolerance to future stress events. Despite the knowledge of changes in metabolites and metabolic pathways during stress, the metabolic imprints as mediators of priming were not well explored. Several experiments revealed that metabolic changes are more sensitive than transcriptional changes, and they constitute early stress responses [171,172]. In summary, plant metabolomic responses to multiple environmental stresses are different from those from a single factor, and stress cross-tolerance or priming is an exciting area for future endeavors [173]. 

### 3.4. Metabolomics of Chemical Agent Treatment and Epigenetic Modifications

In agricultural practice, plants are often treated with chemical agents and plant growth regulators to enhance the defense, yield, and quality of fruits. However, very few reports are available on the effect of these external chemical agents on plant metabolites, which are directly related to defense, quality, and flavor. Aerial sprays of ABA and gibberellin (GA_3_) to grapevine plants cv. Malbec increased the contents of proline, monoterpenes- and sesquiterpenes in the leaves and berries. ABA alone induced the synthesis of anthocyanins in leaves [174]. In *Catharanthus roseus* leaves, exogenous sucrose supplementation elevated the monoterpenoids and indole alkaloids, and their corresponding gene transcript levels [175]. Greenhouse-grown melon treated with the biopolymer-based biostimulant Quik-link showed changes in peptides and lignosulphonates, promoting lateral root growth [176]. 

DNA methylation and histone modification alter gene expression in a heritable fashion without causing any changes in the underlying DNA sequence [177]. The epigenetic modifications altered maize’s pericarp color, anthocyanin, and flavonoid content [178]. In rice, hypomethylation of Rous-associated virus 6 (*RAV6*) promoter in rice *Epi-rav6* mutant showed altered brassinosteroid homeostasis, resulting in altered leaf and grain size [179]. Furthermore, epigenetic modification regulates fruit phenotype by altering a wide range of primary and specialized metabolites in tomatoes. For example, methylation of the SQUAMOSA promoter binding protein-like (SBP-box) genes residing at the epigenetic mutant colorless non-ripening (*Cnr*) locus caused decreases in ethylene and carotenoids, which affected fruit shelf life and quality [180]. The ripening-inhibitor (*rin*) mutant of tomato exhibited reduced amino acids, organic acids, sugars, carotenoids, and ethylene [181]. Histone deacetylases remove the acetyl group from histones on DNA, making the DNA less accessible to transcription factors. Inhibition of histone deacetylase by sodium butyrate during *M. truncatula* seed germination revealed epigenetic up-regulation of antioxidant and polyamine biosynthesis genes, leading to increased antioxidants and seed nucleotide, amino acid, lipid, and carbohydrate metabolism [111]. In another study, metabolomic analysis identified differential buildup of metabolites in *A. thaliana* after induction of the activity of a transcription factor BOLITA involved in plant development [182]. The study provided an epigenetic and accessibility status of the regulatory regions of genes in aerial and root tissues, including the presence or absence of other factors in each tissue. Five differentially accumulated metabolites were found within the flavonoid biosynthesis pathway. The flavonols, kaempferol, quercetin, anthocyanins, and cyanidin were putatively identified as differentially accumulated metabolites, increasing their relative concentration in the roots of plants with higher transcription activity of BOL. Changes in both glucosinolate levels and glucosinolate-related gene expression were also observed. Among the genes that showed differential expression, two that code for nitrilase enzymes, *NITRILASE2* and *4*, have been reported to participate in the degradation of indole glucosinolates. Metabolomics has clearly shown utility in connecting epigenetics to phenotypes/traits. 

### 3.5. Multi-Omics Integration to Analyze Plant Multiple Stress Responses

Multi-omics integration (MOI) has become an integral component of systems biology due to the advances in next-generation high-throughput DNA/RNA sequencing and mass spectrometry-based proteomics and metabolomics [183]. Integrating metabolomics data with other omics data, such as genomics, transcriptomics, or proteomics, contributes to a significant understanding of plant responses to environmental factors/stresses. Over the past few decades, various metabolites have been identified in multiple stress responses and correlated to their transcriptomic and proteomic profiles (Table 1; Supplemental Table S1). MOI approach has been applied to research in humans [184,185], animals [186], and microbes [187,188]. MOI in plants has been relatively complex due to their metabolic diversity, poorly annotated large genomes for non-model species, and the presence of diverse microbiomes with complex interaction networks. Below, we describe recent plant studies integrating metabolomics with other omics in response to single and multiple stresses. 

Transcriptomic and metabolomic analyses of tea plants under drought stress demonstrated that exogenous ABA application significantly reduces drought damage, maintains the balance of primary metabolism, promotes energy storage, and increased production of flavonoids and derivatives to enhance drought tolerance [189]. Using transcriptomic and metabolite analyses, drought tolerance of foxtail millet (*Setaria italica*) during its germination process was correlated with the activation of phenylpropanoid-related pathways, as evidenced by increased amounts of cinnamic acid in germinating seeds under drought [190]. In contrast, p-coumaric acid, caffeic acid, ferulic acid, and sinapic acid were decreased. Many phenylpropanoids and related metabolites have been reported to be allelochemicals and influence germination and plant growth, such as coumaric acid and ferulic acid [190]. Under drought conditions, the drought-tolerant sesame plants up-regulated the expression of genes involved in protein processing, galactose metabolism, hormone signal transduction and down-regulated photosynthesis, based on the changes at both the transcriptomic and metabolomic levels [191]. Transcriptomic and metabolomic responses of *Arabidopsis* leaves exposed to prolonged warming and heat shock treatment increased the rate of transpiration, ROS production, and induction of antioxidant enzymes. Transcription factors, class A1 heat shock factors and dehydration responsive element-binding proteins (DREBs) showed up-regulation under heat shock. Under prolonged warming, basic leucine zipper factors (bZIPs), among other abiotic stress response pathways, showed up-regulation. Additionally, sorbitol showed a considerable increase following prolonged warming, but was not found under heat shock. Carbohydrate conjugates, dihydrosphingosine, methyl-beta-d-galactopyranosic, mannose, and phenyl-beta-d-glucopyranoside were decreased under heat shock [192].

In *Mentha piperita* and *C. roseus*, physiological, biochemical, and metabolomic variations following heat and water stress imposition (alone and combined) increased the accumulation of osmolytes and specialized metabolites at the early and late growth stages. Drought and/or heat stress triggered the accumulation of osmolytes (proline, sugars, glycine betaine, and sugar alcohols, including inositol and mannitol), with maximum accumulation in response to the combined stress. Due to drought and heat stress, total phenol, flavonoid, and saponin contents decreased. Levels of other specialized metabolites (e.g., tannins, terpenoids, and alkaloids) increased under stress, with the maximal accumulation under the combined heat and drought stresses [190,193]. Under high night temperature (HNT), a highly activated TCA cycle and concomitantly increased levels in pathways branching off from the TCA cycle into amino acid and polyamine biosynthesis were determined. Transcript and metabolite profiling of leaves from six rice cultivars under HNT identified six genes as central for the HNT response. They encode proteins involved in transcription regulation and biosynthesis of specialized metabolites. Sensitive cultivars showed specific changes in ABA signaling and cell wall-related genes. Metabolite profiles revealed a highly activated TCA cycle under HNT and concomitantly increased levels in primary metabolism, including TCA cycle, sugar, and amino acid metabolism, which were corroborated by the corresponding enzyme activity changes [104]. Integrating metabolomic and transcriptomic analysis revealed significant differences in cutin biosynthesis between two *Capsicum chinense* genotypes, and assessed the roles of cutin lipids in postharvest water loss [194]. In analyzing the effects of salt stress on *Zygophyllum brachypterous*, *Z. obliquum*, and *Z. fabago*, transcriptomics revealed significant changes in two branched-chain aminotransferase (BCAT) genes among the 11 differentially expressed genes, significantly enriched in valine, leucine, and isoleucine biosynthesis [195]. Additionally, various metabolite intermediates of the TCA cycle, such as fumaric acid, malic acid, and citric acid, were decreased after prolonged warming and heat stress. Furthermore, the pantothenate and CoA biosynthesis pathways were enriched after salt stress, which was consistent with the KEGG pathways enriched according to the transcriptomics data. It is possible that the BCATs may affect the pantothenate and CoA biosynthesis pathways to regulate salt tolerance of *Zygophyllum* species. This may constitute a newly identified pathway through which plants respond to salt stress.

Quantitative phosphoproteomic and metabolomic approaches were employed to identify changes in phosphoproteins and metabolites in soybean roots treated with rhizobia inoculation and salt stress [196]. Rhizobia-inoculation repressed the phosphorylation of two transcription factors, GmMYB173 and GmMYB183, which control the expression of GmCHS5 and GmCYP81E11, enzymes involved in flavonoid synthesis. Concomitantly, inoculation of soybean roots by rhizobia resulted in significant increases in the levels of 19 flavonoids. When the rhizobia-inoculated roots were further treated with salt stress, the accumulations of 32 flavonoids were significantly increased, thereby helping the plants adapt to salt stress [196]. 

Cold-susceptible and the cold-resistant species of *Momordica charantia* (bitter gourd) were investigated for their cold-adaptation mechanism [197]. After cold treatment, the levels of malondialdehyde (MDA), hydrogen peroxide (H_2_O_2_), proline, antioxidant enzymes, metabolites, and gene expression were analyzed. The results showed that the low-temperature stress damaged the growth of the cold-sensitive seedlings but not the cold-resistant seedlings. Enhanced expression of transcriptional factors, ROS-related enzymes, and increased levels of sugar, fatty acid, and amino acid enabled cold tolerance in the seedlings [197]. In *Arabidopsis* plants exposed to dehydration, high salinity, extended darkness, cold, or heat, combined analysis of transcriptomic and metabolomic datasets revealed amino acids such as proline, arginine, glutamine, and GABA act as compatible osmolytes, precursors for specialized metabolites, or storage forms of organic N [198]. Metabolomics and transcriptomics of soybean leaves revealed relevant components in different drought and heat scenarios and the relationships between molecular players of the stress responses [199]. Most single stress responses are maintained in combined drought-heat stress, and drought and heat stress interact at the transcript and metabolite levels. It is interesting to note that the drought response dominated the heat response in the combined stress. The interaction effects between drought and heat are related to protein folding, flavonoid biosynthesis, and growth inhibition, which were enhanced, reduced and induced, respectively, by the combined stress. Moreover, the datasets of comprehensive gene expression, targeted and untargeted metabolomics serve as a resource to query the functions of candidate genes and/or metabolic features toward investigating specific response mechanisms, with the ultimate goal of sustaining crop production in the changing climate conditions.

### 3.6. Single-Cell and Spatial Metabolomics

**Figure 4:**
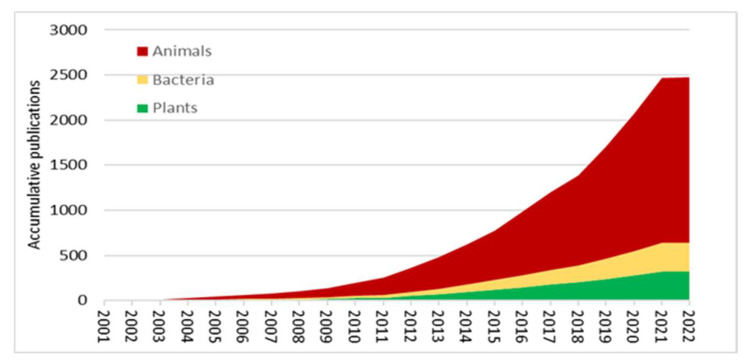
Publications generated on single-cell metabolomics in the past 20 years. Publication numbers were obtained from PubMed search using single-cell metabolomics as keywords. Please note that 2022 is for the first five months only.

Recently, new technologies have been geared toward experimental tools that enable single-cell proteomics, metabolomics, and spatially resolved transcriptomics at the single-cell level. Some of these tools have shown utility in algae, fungi, and plants over the past few years [72,200]. While single-cell metabolomics is advanced in animal cells, technical challenges need to be overcome in plants before it can be broadly applied. Plant single-cell metabolomics involves many dynamically changing metabolites with a wide range of concentrations influenced by different environmental factors (Figure 4).

**Figure 5:**
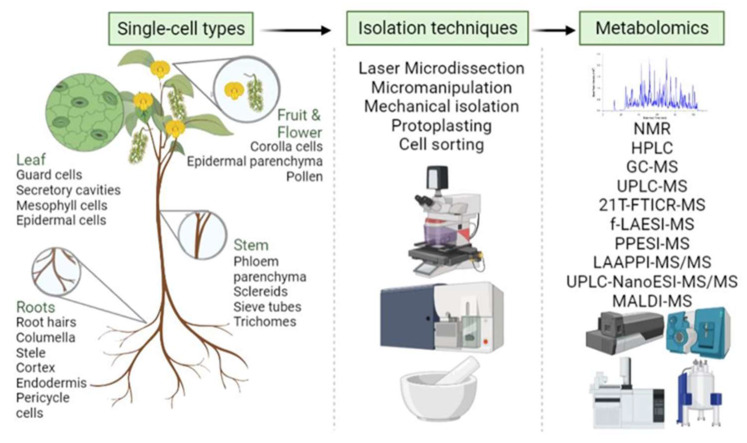
Single-cell metabolomics workflow from different cell types (e.g., epidermal cells, guard cells, root hairs, floral organ cells, root tip stem cells, root nodule cells, hairy root culture cells, in vitro callus cultures, parenchyma and sclerenchyma cells, mesophyll and bundle sheath cells, and inner endodermal cells), and organelles including apoplast, chloroplast, and mitochondria. They were isolated from plant tissues using methods including laser microdissection, laser-ablation, laser capture microdissection-liquid vortex capture, micromanipulation, mechanical isolation, protoplasting, pressure probe, and cell sorting. NMR: Nuclear magnetic resonance; HPLC: High performance liquid chromatography; LMD LC-MS: Laser Microdissection liquid-chromatography mass spectrometry; LAAPPI: Laser ablation atmospheric pressure photoionization; GC: Gas chromatography; LAESI: Laser ablation electrospray ionization; picoPPESI: picolitre pressure-probe electrospray-ionization; fLAESI: Optical fiber-based laser ablation electrospray ionization; 21TFTICR-MS: 21 tesla (T) Fourier transform ion cyclotron resonance MS; LSC-MS: Live single-cell MS; UPLC: Ultra performance liquid chromatography; NanoESI: Nano electrospray ionization; MALDI: Matrix-assisted laser desorption and ionization.

As single-celled photosynthetic organisms, algae have been the focus for producing high-value metabolites, food, and fuel. A robust high throughput extraction method was developed to extract chlorophyll, lipids, metabolites, proteins, and starch simultaneously from a single sample of the green alga *Chlamydomonas reinhardtii* [201]. A protocol for depositing, isolating, and characterizing single cells (Figure 5) by laser capture microdissection has been developed, enabling the online metabolic profiling of cell sections in *Allium cepa* (red onion) [202].

The combination of fiber-based laser ablation electrospray ionization (fLAESI) with 21 T Fourier transform ion cyclotron resonance MS (21TFTICR-MS) for in situ single-cell metabolic profiling was employed in soybean root nodules [76]. Single plant cells infected by bacteria were sampled directly from the tissue without cell manipulation through mid-infrared ablation with a fine optical fiber tip for ionization by fLAESI. Ultra-high performance 21T-FTICR-MS enabled simultaneous capture of delicate isotopic structures for 47 known and 11 unknown metabolites, thereby elucidating their elemental compositions from single cells and providing information on metabolic heterogeneity in the cell population. Wada et al. [203] performed picolitre pressure-probe-ESI MS to directly determine metabolites in growing inner endosperm cells of intact seeds produced under HNT conditions, combining with 13C feeding and water status measurements, including an in situ turgor assay. Their studies revealed the accumulation of active solute, including UDP-glucose, UDP-D-xylose (arabinose), and UDP, was caused by inhibition of wall and starch biosynthesis, leading to the partial arrest of cell expansion in the inner endosperms under the HNT conditions.

Single-cell metabolomics in plant cell cultures has been extensively carried out. Abiotic elicitation by UV-C radiation and melatonin significantly enhanced the biosynthesis of polyphenols and flavonoids in the callus cell culture of *L. sativum* [204]. Melatonin appeared to be a more effective elicitor than UV-C, leading to increases in the specialized metabolites, e.g., chlorogenic acid, kaempferol, and quercetin. Coupling data-processing software with capillary zone electrophoresis (CZE)-MS data acquisition has enabled comprehensive metabolomic profiles from *Lobelia cardinalis* cell cultures [205]. The primary alkaloid lobinaline and several putative “lobinaline-like” molecules were significantly increased in the *L. cardinalis* hairy root cell cultures in response to N-methyl-4-phenylpyridinium (MPP^+^), which is an active metabolite of the neurotoxin N-methyl-1,2,3,6-tetrahydropyridine. The CZE-MS has shown utility in separating and identifying novel bioactive metabolites from plant cell cultures [205]. In another study, Wahyuni et al. detected four metabolites with antifungal, herbicidal, and insecticidal properties, namely, pelargonic acid, decanoic acid, hexadecanoic acid, and a new terpenoid by GC-MS and thin-layer chromatography of callus cultures of *Sonchus* *arvensis* [206]. Please refer to Table 2, for the comprehensive list of single-cell metabolomics studies in protists and plants and the advancements in the single-cell and spatial metabolomics methods.

Over the past decade, MALDI MSI has been popular in spatial metabolite analysis in animal cells [220]. However, its application in plant cell imaging is still in the early stage. The sensitivity with the single-cell resolution is a great challenge. Microarray-based sample preparation workflow for MALDI permits single-cell sensitivity and high-throughput analysis of lipids and pigments in single algae cells. The microarray targets have individual cells in 1430 different spots that allow the cells to be lysed individually without cross-contamination. The mass spectra unveiled information about the relative composition of more than 20 different lipids/pigments in each cell within the population [221]. In another study, cytotoxicity of three herbicides on *C. reinhardtii* was determined by analyzing the lipid variation upon herbicide exposure using MALDI-MS [72]. At lower herbicide concentrations, digalactosyldiacylglycerol showed a rapid decrease in abundance, while several other lipids displayed moderate increases. Metabolomics was also conducted in other algae, *Euglena*, Haematococcus, Nannochloropsis and Zygnema, to demonstrate the differential metabolic profiles in response to light/dark, melatonin and N starvation [168,207,208,209,210,222].

Single-cell metabolomics unraveled the complexity of intercellular localization of specific terpenoid indole alkaloids using MSI in *C. roseus* [68]. They were found to localize in the epidermal cells, and some indole alkaloids (including serpentine and vindoline) were localized in idioblast cells. Their accumulation increases in laticifer cells as the leaf expands. Using laser microdissection (LMD), *Arabidopsis* epidermis, cortex, vascular bundles, and pith of flowering stems were isolated. LC-MS/MS metabolomics revealed spatiotemporal changes in plant hormones in a region and tissue-specific manner [211]. Carbon-based nanomaterials have potential applications in agriculture. However, the physiological and molecular mechanisms underlying single-walled carbon nanohorn (SWCNH)-mediated plant growth remain unclear. Metabolomic analyses revealed that SWCNHs altered the levels of sugars, amino acids, and organic acids, suggesting that SWCNHs reprogrammed carbon/nitrogen metabolism in plants [170]. SWCNTs also regulate plant growth and development by increasing the levels of several specialized metabolites such as flavone, anthocyanins, flavonol, and isoflavone of tea leaves under drought stress; transcriptomic analyses of their biosynthetic genes further supported these results [189]. These multi-omics approaches serve as examples for studying the effects of nanomaterials in plants. 

In another study, 200 targeted single cells of *Allium cepa* were analyzed using a LAESI dual optical microscope source coupled to an FT-ICR for high-mass-resolution and high-mass-accuracy metabolomics [215]. This emerging method allowed for analyzing the metabolites present in *A. cepa* epidermal cells with confident structural identification. The authors also conducted in situ spatial metabolomics via imaging of *Fittonia argyroneura* leaves and identified chemical species specific to the physical structures in the leaves. These studies demonstrate the potential application in future spatial metabolomics of single cells. It requires minimal sample preparation and utilizes water for in situ analysis, among the many other benefits. Infrared laser ablation atmospheric pressure photoionization MS (LAAPPI-MS) imaging was employed to conduct metabolite profiling at various layers of *Arabidopsis* leaf tissues, ranging from trichomes to veins. This demonstrated that the distributions of different flavonol glycosides, fatty acids, fatty acid esters, galactolipids, and glycosphingolipids varied significantly between the different substructures in the leaves [212]. 

A combined approach of meta-transcriptomics and metabolite profiling in environmental samples of *Zygnema* sp., collected from arctic Svalbard to understand the spatial organization of *Zygnema* mats, revealed that the top layer is very active and appeared to act as a sunshade for bottom layers in a meltwater streamlet. GC-MS-based metabolome profiles of the two layers indicated that 15 metabolites accumulated in the top layer were mostly sugars and sugar alcohols (e.g., glucose, maltose, mannose, and sorbitol). These accumulated metabolites may be attributed to the exposure of the the upper layer to high photosynthetically active radiation and ultraviolet radiation [211]. Spatial-resolution targeted metabolomics in a single tea leaf showed quantitative variation within-leaf for distinguished isomeric metabolites such as kaempferol 3-glucoside and kaempferol 3-galactoside, and quercetin 3-glucoside and quercetin 3-galactoside [223]. These studies lead to a better understanding of the cellular and spatial distribution of metabolites in different plants and biological processes and how metabolites play a role in plant growth, development, and interaction with the environment. 

The molecular dynamics of plant cells with different layers of information exhibit diversity in the regulatory mechanisms [224,225]. Despite our knowledge of apoplast involvement in cell growth and stress responses, its dynamics are still poorly known. Figueiredo et al. [218] developed the vacuum-infiltration-centrifugation method that allows a simultaneous extraction of grapevine apoplastic proteins and metabolites from leaves, which are compatible with proteomic and metabolomic analyses. NanoLC-MS/MS-based proteomics identified more than 700 proteins with diverse biological functions. The metabolomic profile through FT-ICR-MS included 514 metabolites covering a broad spectrum of molecular classes. Studies on single-cell metabolomics and transcriptomics determined that ray parenchymal cells function in the lignification of upright tracheids by biosynthesis of monolignols in lignifying xylem cells of *Picea abies* [217]. Transcriptomic analysis revealed that among the genes involved in the processes typical for vascular tissues, cell wall biogenesis enzymes were upregulated in both ray cells and tracheids. Additionally, the genes in monolignol and shikimate biosynthesis pathways were equally expressed in both cell types. In situ single-cell metabolomics by picoPPESI MS discovered monolignols and their glycoconjugates in ray cells and tracheids, indicating that the biosynthetic route for monolignols may be active in tracheids and parenchyma ray cells. The data supports the premise that in developing xylem, ray cells produce monolignols that contribute to the lignification of tracheid cell walls.

Cotton fiber is a vital plant single-cell model and a textile application material. However, its color formation is poorly understood, although it is known to be regulated by environmental signals. Transcriptomic and metabolomic studies revealed complex pathways involved in early initiation and late metabolic pathways, the effect of photo signals on fiber color formation. They characterized fiber color early initiation and late accumulated metabolites in different lighting conditions [216]. This study on single-cell fiber provides new insight into the molecular regulatory mechanisms and biochemical bases underlying the photo-induced fiber color formation in cotton. Overall, these studies improved understanding of metabolite functions at different spatial definitions, including the single-cell level. Although single cell metabolomics is still challenging, recent advances in MS-based technologies (e.g., LAESI and TIMS) will ensure significant progress in the near future.

## 4. Challenges and Future Perspectives

Over the past two decades, great advances in plant metabolomics have been achieved. They have enabled discovery of important molecules linked to genetic pathways associated with crop yield and stress tolerance. They also helped to identify molecular markers for marker-based breeding or crop engineering for improving yield, quality, and stress resilience [226,227]. We have envisaged various advances in analytical technologies ranging from profiling targeted metabolites to an array of global metabolites. The integration with other ‘omics data, such as epigenetics, transcriptomics, and proteomics, uncovered different regulatory mechanisms and important metabolites specific to crop traits. It has also led to the discoveries of new signaling and metabolic networks and the establishment of predictive models and, undoubtedly, these systems biology approaches have proven effective. However, an enormous amount of work is still required to characterize the functional implications of different metabolites in signaling and metabolism, as well as in single-cell or single-cell types. 

One of the limitations of plant metabolomics is the size and availability of the databases and libraries. With over one million plant metabolites estimated to date [81], only 50,048 metabolites from 20,741 plant species, have records from the most extensive plant metabolite database KNApSAcK. At the same time, only 4806 metabolite records in PlantCyc and 2854 in KEGG are available in the metabolic pathways. Although public tandem mass spectral libraries, such as mzCloud, NIST, and METLIN, enable the identification of thousands of metabolites, they are not targeted to plant metabolites. In-house spectral libraries have more specificity for plants [209], but there is no convenient platform for researchers to share their in-house libraries. Additionally, the libraries that have been created based on a specific vendor’s mass spectrometer system are limited to that vendor’s system and not transferrable to other systems. Presently, about two thousand plant metabolite standards are available, which allow the expansion of plant metabolite spectral libraries. 

Another grand challenge is multi-omics integration, modeling, and simulation. The current limitations can be facilitated by increasing and integrating computing power for data processing, machine learning, and artificial intelligence. Developing user-friendly community-wide software packages would enable the generation and testing of specific mechanistic hypotheses of the molecular processes underlying critical biological functions. The integrated collaboration of biologists, chemists, and bio-informaticians will yield novel approaches and solutions for analyzing interactions between plants and various biotic and abiotic stresses. Characterization of the metabolic heterogeneity in cell populations requires the analysis of single cells. Most current methods in single-cell analysis rely on cell manipulation, potentially altering the abundance of metabolites in individual cells. A small sample volume and the chemical diversity of metabolites are additional challenges in single-cell metabolomics. Applying single-cell techniques to microbial and fungal species, and plants, would enable a greater understanding of how plants and microbes interact in a competitive or symbiotic relationship. Isotope labeling offers an excellent opportunity to differentiate plant metabolites from microbes [142]. Advances in single-cell technologies will have exciting and far-reaching impacts when widely applied to plants, fungi, and microbes and will be transformative for both our improved understanding of environmental biology and for trait engineering in food security, bioenergy, and biomaterials.

## Figures and Tables

**Table 1:** Summary of plant metabolomics studies of different model species using various high-throughput metabolomics technologies *.

Plant	Stress	Method	Tissue	Type of Metabolites	Refs
*A. thaliana*	Heat shock	GC-MS	Root and shoot	Glyceric acid, maltose, asparagine, glutamine, glycine, and trehalose	[91]
		LC-MS/MS	Leaf	Phenylalanine-derived metabolites	[92]
	Drought	LC/UPLC-MS/MS; GC-MS		Hormones, MGDG, DGDG, SQDG, PC, PE, PS, PI, and TAG	[93]
	Heat primed, heat shock	UPLC-MS/MS; HILIC/UPLC-MS/MS		Amino acid, carbohydrate, lipid, nucleotide, 2-isopropyl malate, dihydrokaempferol, putrescine, 2-hydroxy laurate, glycerol 3-phosphate, glutathione, ascorbate, tocopherol, and GPC	[94]
	High light, cold	GC-TOF MS; LC-MS/MS		Citrate, gluconic acid, hexose, amino acid, organic acid, sugar	[95]
		GC-TOF-MS		Sucrose phosphate, starch, serine, raffinose, pyruvate, malate, and proline	[96]
		GC-TOF-MS		Sucrose, glucose, and fructose	[97]
	Bacteria	UPLC-MS/MS		Hormone and redox metabolites	[98]
	*Genetic* *modification of AtTSPO*	^1^H-NMR; HPTLC	Seed and leaf	Fatty acids, lipids, sterols, esters, TAG, FFA, DAG, and starch	[99]
	Drought, heat	FT-ICR-MS; GC-TOF-MS	Leaf	Sucrose, sorbitol, coniferyl alcohol, cinnamyl alcohol, and fatty acids	[100]
*Brachypodium distachyon*	Drought	GC–MS	Leaf	Fatty acid, malic acid, amino acids	[101]
*Lotus japonicus*	*Agrobacterium*	LC-IonTrap-MS/MS; NMR	Flower buds and petals	Flavonoids, quercetagetin, gossypetin	[102]
*Physcomitrella patens*	Salt, cold, abscisic acid	GC-MS	Protonema tissue	Sugar, amino acid, and organic acid	[103]
	High night temperatures	GC-TOF-MS	Leaf	Amino acids, sugars, organic acids, phenylpropanoids, phosphates, and polyhydroxy acids	[104]
*Oryza sativa*	Drought	GC-MS; LC	Leaf	Citric and aconitic acids, benzoic acid, carbohydrates, proline, norvaline, GABA, benzoic acid, TCA cycle acids, and sugars	[105]
	Salinity	GC-TQMS	Leaf and root	Mannitol, sugar, and organic acid	[106]
*Hordeum vulgare*	Salinity	LC-MS/MS; MALDI–MSI	Root	PC, fatty acyls, glycerophospholipid, glycerolipid, prenol lipid, polyketide, sphingolipid, DAG, TAG, and SQDAG	[107]
		GC-MS; LC-MS/MS	Root and leaf	Phytohormones and chlorophyll	[108]
	Drought, bacteria	LC-MS/MS	Leaf	Flavonoid, auxin, flavonol, flavanone, anthocyanin, and hormones	[109]
*Medicago sativa; M. truncatula*	Drought	LC-TQMS	Leaf and root	Flavonoid, carbohydrate, abscisic acid, and proline	[110]
*M. truncatula*	Drought, *Fusarium* *oxysporium*	LC-MS/MS		Organic acid, sugar, citrate, isocitrate, and tetrahydroxychalcone	[111]
	Histone deacetylase inhibitor	LC-MS/MS; GC-MS	Seed and seedling	Amino acid, lipid, and carbohydrate; saccharopine, UDP-glucose/UDP-galactose, 1-linolenoylglycerol, 1-linoleoyl-GPI 182, creatine, and N-acetylglutamine	[112]

* The detailed list of metabolomics studies in other plants is provided in Supplementary Table S1. GC-MS: Gas chromatography-mass spectrometry; LC-MS/MS: Liquid chromatography-tandem mass spectrometry; UPLC: Ultra performance LC; HILIC: Hydrophilic interaction chromatography; HPTLC: High-performance thin-layer chromatography; TQMS: Triple quadrupole MS; 1^H^NMR: proton nuclear magnetic resonance; FT-ICR-MS: fourier-transform ion cyclotron resonance MS; MALDI-MSI: matrix-assisted laser desorption ionization MS imaging; IonTrap-MS/MS: ion trap MS/MS; GC-TOF-MS: GC-time of flight MS; MGDG: Monogalactosyldiacylglycerol; DGDG: Digalactosyldiacylglycerol; SQDG: Sulfoquinovosyldiacylglycerol; PC: phosphatidylcholine; PE: phosphatidylethanolamine; PS: phosphatidylserine; PI: phosphatidylinositol; TAG: Triacylglycerides; GPC: Glycerophosphorylcholine; SQDAG: Sulfoquinovosyl diacylglycerol; FFA: Free fatty acids; DAG: Diacylglycerol; GABA: Gamma aminobutyric acid; AtTSPO: *A. thaliana* translocator protein.; UDP: Uridine diphosphate.

**Table 2:** Single-cell and spatial metabolomics in algae, *A. thaliana*, and other plants.

Name	Treatment	Instrument	Cell Type	Metabolites	Refs
*Chlamydomonas reinhardtii*	Herbicide	MALDI-MS	Single-cell	Lipids; DGDG, TAG, DGTS	[72]
	Light/dark	GC-TOF-MS; UPLC	Single-cell	Lipids, nucleic acids, intermediates of glycolysis, TCA metabolites, polyamines	[201]
*Euglena gracilis*	Light/Dark	GC-MS	Chloroplast	Amino acids, lipid metabolites	[207]
*Haematococcus pluvialis*	Melatonin	LC-MS	Single-cell	Carotenogenic, astaxanthin, and lipids	[208]
	High light, fulvic acid, and N starvation	LC-MS/MS	Single-cell	Astaxanthin, carbohydrates, lipids	[169]
*H. pluviali, Coscinodiscus granii*	None *	LDI-HR-MS	Cell wall, single-cell	Photosynthetic pigments	[209]
*Zygnema* sp.,	None *	GC-MS	Single-cell	Chlorophylls	[210]
*Fucus vesiculosus*	Various seasons	UPLC-MS^N^	Single-cell	Chlorophylls, phlorotannin, lipids, and carotenoids	[77]
*A. thaliana*	None *	LMD- LC-MS/MS	Epidermis, cortex, vascular bundles and pith of flowering stem	IAA, JA	[211]
	None *	LAAPPI-MS/MS	Trichome, single cells, interveinal lamina	Flavonol glycosides, fatty acids, fatty acid esters, galactolipids, and glycosphingolipids	[212]
	Hormone, *Haloperonospora*	NanoLC ESI-MS/MS	Mesophyll, epidermal, and stomatal guard cells	Phytohormones	[200]
	Single-walled carbon nanohorn (SWCNH)	GC-MS	Root tip, stem cells	Auxin, serine, methionine, 3,5,7-trihydroxy-4′-methoxy flavone, citraconic acid, hypoxanthine, cellotetraose, 3,4′,5,6,7-methoxyflavone, serotonin, 2,3,4-tri methoxy mandelic acid, epicatechin, furfuryl alcohol, glycolic acid, and ß-sitosterol	[213]
	Dark	GC-MS	Leaf mitochondria	Chlorophylls, proline	[214]
*Allium cepa*, *Chlamydomonas reinhardtii*	None	LMD-LVC-MS/MS	Epidermis of *A. cepa* and microalgal cells		[202]
*Allium cepa*, *Fittonia* *argyroneura*	None	LAESI-MS/MS	*F. argyroneura* leaves, epidermal layers of *A. cepa*	Acids, carbohydrates, catechol, phthalide, lysine	[215]
*Catharanthus roseus*	None	MSI	Leaf spatial imaging	Terpenoid indole alkaloids	[68]
*Glycine max*	Soil bacteria, *Bradyrhizobium japonicum*	21T-FTICR-MS; fLAESI-MS	Root nodule cells	lipids, oligosaccharides, and soyasaponins	[76]
*Gossypium* *hirsutum* L.	Shading	UPLC-MS/MS	Cotton fibers cells	Amino acids and derivatives, phenylpropanoids, nucleotides and derivatives, lipids, organic acids	[216]
*Lobelia cardinalis*	None	CZE-MS	Hairy root cell cultures	Alkaloids	[205]
*Oryza sativa*	High night temperature	PPESI-MS	Inner endosperm cells	Sugars; malic acid, glutamic acid, ascorbic acid, and Hexose	[203]
*Picea abies*	None	picoPPESI-MS/MS	Parenchymal ray cells, tracheid of the xylem	Organic acids, sugars, most amino acids, glutathione, and abietic acid; coniferin, p-coumaryl alcohol 4-glucoside, and quinic acid	[217]
*Sonchus arvenis*	Dolomite	GC-MS; TLC	Leaf callus, sclerenchyma, parenchyma cells	Pelargonic acid, decanoic acid, and hexadecanoic acid	[206]
*Vitis vinifera*	None	NanoLC-MS/MS	Leaf apoplast	Lipids, phenolic metabolites, and carbohydrates	[218]
*Zea mays*	None	MALDI-MSI; GC-MS	Thylakoid membranes, mesophyll, and bundle sheath cells	600 metabolites: primary amines, carbonyl groups, carboxylic acids	[219]

* None indicates no treatment, and the plant metabolites were studied under normal growth conditions. Please refer to Table 1 for some abbreviations. Methods: MALDI-MS: Matrix-assisted laser desorption ionization-mass spectrometry; GC-TOF-MS: Gas chromatography time-of-flight MS; UPLC: Ultra performance liquid chromatography; GC: Gas chromatography; LC-MS/MS: Liquid chromatography tandem MS; LDI-HR-MS: Laser desorption ionization high-resolution MS; LMD: Laser microdissection; LAAPPI: Laser ablation atmospheric pressure photoionization; NanoESI: Nanoelectrospray ionization; LMD-LVC-MS/MS: LMD-liquid vortex capture tandem MS; LAESI: Laser ablation electrospray ionization; MSI: Mass spectrometry imaging; 21T-FTICR-MS: 21 tesla (T) Fourier transform ion cyclotron resonance MS; fLAESI: Optical fiber-based laser ablation electrospray ionization; CZE-MS: Capillary zone electrophoresis MS; PPESI: Pressure-probe electrospray-ionization; picoPPESI: Picolitre pressure-probe electrospray-ionization; TLC: Thin layer chromatography; NanoLC: Nanoflow LC; QTOF: Quadrupole time of flight; MSn: Multiple stages of MS; DGTS: diacylglyceryltrimethylhomo-Ser; DGDG: digalactosyldiacylglycerol; TAG: triacylglycerol; TCA: Tricarboxylic acid cycle; JA: Jasmonates; IAA: indole-3-acetic acid; UV: ultraviolet; IAA: indole-3-acetic acid.

## Data Availability

Not applicable.

## Supplementary Materials

The following supporting information can be downloaded at: https://www.mdpi.com/article/10.3390/ijms23136985/s1. References [228,229,230,231,232,233,234,235,236,237,238,239,240,241,242,243,244,245,246,247,248,249,250,251,252,253,254,255,256,257,258,259,260,261,262,263,264] are cited in Supplementary Materials.
